# Evaluation of antimotility effect of *Lantana camara L. *var. acuelata constituents on neostigmine induced gastrointestinal transit in mice

**DOI:** 10.1186/1472-6882-5-18

**Published:** 2005-09-17

**Authors:** Lenika Sagar, Rajesh Sehgal, Sudarshan Ojha

**Affiliations:** 1 Department of Biochemistry, Panjab University, Chandigarh – 160 014 India

## Abstract

**Background:**

*Lantana camara *L. (Verbenaceae), a widely growing shrub which is toxic to some animal species, has been used in the traditional medicine for treating many ailments. The purpose of the present study was to evaluate the antimotility effects of *Lantana camara *leaf constituents in mice intestine.

**Methods:**

Evaluation of antimotility activity was done in intestine of mice treated with *Lantana camara *leaf powder, *Lantana camara *methanolic extract (LCME), lantadene A, neostigmine and neostigmine + LCME. Neostigmine was used as a promotility agent. Intestinal motility was assessed by charcoal meal test and gastrointestinal transit rate was expressed as the percentage of the distance traversed by the charcoal divided by the total length of the small intestine. The antidiarrheal effect of LCME was studied against castor oil induced diarrhea model in mice.

**Results:**

The intestinal transit with LCME at a dose of 500 mg/kg was 26.46% whereas the higher dose (1 g/kg) completely inhibited the transit of charcoal in normal mice. The % intestinal transit in the neostigmine pretreated groups was 24 and 11 at the same doses respectively. When the plant extracts at 125 and 250 mg/kg doses were administered intraperitonealy, there was significant reduction in fecal output compared with castor oil treated mice. At higher doses (500 and 1000 mg/kg), the fecal output was almost completely stopped.

**Conclusion:**

The remarkable antimotility effect of *Lantana camara *methanolic extract against neostigmine as promotility agent points towards an anticholinergic effect due to *Lantana camara *constituents and attests to its possible utility in secretory and functional diarrheas and other gastrointestinal disorders. This effect was further confirmed by significant inhibition of castor oil induced diarrhea in mice by various doses of LCME.

## Background

Diarrhea is one of the most prevalent human disorders and understandably its remedy occupies a special place in the annals of medicine [1]. Neurohormonal mechanisms, pathogens, malnutrition, chronic diseases and drugs can alter gastrointestinal physiology resulting in changes in either secretion or absorption of fluid by the intestinal epithelium. Altered motility contributes in a general way to this process, as the extent of absorption, by and large, parallels transit time. Prokinetic agents, organophosphate pesticides, nerve gases, surgery, irritation bowel syndrome, collagen vascular disease and diabetes are some of the pathophysiological conditions that may alter intestinal motility and transit time. Antimotility compounds such as diphenoxylate, loperamide, opium alkaloids, anticholinergics etc. have been tried against diarrheal disorders but often with side effects after prolonged use [2].

Acetylcholine, the vagal neurotransmitter, enhances and atropine, a known anticholinergic agent decreases intestinal motility and secretion. Although various derivatives and congeners of atropine (such as propantheline, isopropamide and glycopyrrolate) have been advocated in patients in peptic ulcer or with non-specific diarrhea, the prolonged use of such agents is limited by other manifestations of parasympathetic inhibition such as dry mouth and urinary retention [3]. There is, thus, a need for identifying new compounds and evaluating their antimotility activity and developing these as selective inhibitors that decrease gastric secretion and intestinal motility at doses that have minimal anti-cholinergic effects at other sites and are completely free from other adverse effects [4].

*Lantana camara *L. (Verbenaceae) is one of the most prevalent and noxious weeds causing hepatotoxicity in grazing animals [5,6]. *Lantana *poisoning causes obstructive jaundice and within a few hours of browsing upon its foliage, animals go off-feed and become severely constipated within 48 h [7]. On the contrary, *Lantana *plant has been reported to possess a number of medicinal properties [8,9]. Some metabolites isolated from their leaves possess antitumor activity [10], antithrombin activity [11], anti-inflammatory, antinociceptive and antipyretic activity [12].

Present investigations were planned to study the effect of *Lantana camara *leaf powder, *Lantana camara *methanolic extract (LCME) and lantadene A administration on mice intestinal motility using neostigmine as a promotility agent. Antidiarrheal effect of LCME was studied using castor oil induced diarrhea in mice.

## Results

The percent intestinal transit was increased significantly with neostigmine, but was decreased significantly by all concentrations of LCME and lantadene A. The intraperitoneal administration of the LCME (125, 250 & 500 mg/kg) alone decreased the percent intestinal transit significantly. However, with a LCME dose of 1000 mg/kg, intestinal transit was nearly abolished. Leaf powder (1% of feed) feeding for 10 days reduced the % intestinal transit by 34.78. Inhibition in % intestinal transit by 85 and 170 mg/kg lantadene A was 39.47 and 27.34 respectively. The prokinetic effect of neostigmine was opposed by both the doses (1.0 g/kg and 500 mg/kg) of LCME and % intestinal transit was reduced to 11 and 24 respectively (Table 1).

**Table 1:** Effect of *Lantana camara *leaf powder, LCME, lantadene A and neostigmine on small intestinal transit in mice.

**Treatment**	**Total length of intestine (cm)**	**Distance traveled by charcoal (cm)**	**% Intestinal Transit**
CMC Control	45.66 ± 6.24	21.66 ± 0.57	47.25 ± 6.10
Leaf Powder (1% for 10 days)	46.50 ± 6.19	15.50 ± 7.70	34.78 ± 3.52**
LCME (1 g/kg)	50.00 ± 0.90	0.50 ± 0.01	1.00 ± 0.01***
LCME (500 mg/kg)	53.66 ± 6.02	11.00 ± 6.02	26.46 ± 6.82***
LCME (250 mg/kg)	48.30 ± 1.36	15.30 ± 0.51	31.74 ± 1.49***
LCME (125 mg/kg)	49.00 ± 0.89	19.00 ± 3.58	38.67 ± 6.60***
Lantadene A (85 mg/kg)	44.00 ± 7.92	18.01 ± 7.37	39.47 ± 10.05**
Lantadene A (170 mg/kg)	47.00 ± 4.73	13.00 ± 3.03	27.34 ± 4.58**
Neostigmine (1 μg/kg)	49.00 ± 4.69	34.33 ± 9.15	69.30 ± 12.47***
Neostigmine (1 μg/kg)+LCME (1 g/kg)	48.00 ± 3.00	5.33 ± 0.76	11.10 ± 1.14***
Neostigmine (1 μg/kg)+LCME (500 mg/kg)	50.50 ± 0.70	12.00 ± 1.40	24.00 ± 2.19***

**Statistical analysis**: Values are mean ± S.D. of 6 observations in each group. Values having P < 0.01 were considered significant. **P < 0.01, ***P < 0.001 as compared to control.

Diarrhea was apparent in all the animals of control group 45 minutes after the administration of castor oil and fecal count was taken for 4 h. A marked reduction in the number of defecations over 4 h was observed with the i.p. administration of all doses of LCME. The animals of group 4 and 5 appeared to be completely constipated, where as those of group 2 and 3 showed a significant reduction in defecations as compared to control group (Table 2).

**Table 2:** Effect of *Lantana camara *methanolic extract (LCME) on castor oil-induced diarrhea in mice.

**Treatments**	**Mean defecation in 4 h**
Castor oil + saline (2 ml/kg)	24 ± 3.10
Castor oil + LCME (125 mg/Kg)	9 ± 1.18***
Castor oil + LCME (250 mg/kg)	9 ± 2.06***
Castor oil + LCME (500 mg/kg)	1 ± 0.05***
Castor oil + LCME (1000 mg/kg)	Completely constipated

**Statistical analysis**: Values are mean ± S.D. of 4 observations in each group. Values having P < 0.01 were considered significant. ** P < 0.01, *** P < 0.001 as compared to control.

## Discussion

The pathophysiological mechanisms underlying the loss of intestinal fluid in diarrhea have been the subject of much debate for decades [17]. Diarrhea may be caused by an increase in osmotic load within the intestine, excessive secretion of electrolytes and water into the intestinal lumen, exudation of protein and fluid from the mucosa, infection and inflammation; and altered intestinal motility, resulting in rapid transit [18]. In most instances, multiple processes are simultaneously affected involving several factors, a particular factor becoming a dominant player in a given environment, however, motility and/or secretory disturbances usually remain a common denominator in most cases [2]. The mucosal lining of the gastrointestinal tract is provided with an extensive nerve supply from the enteric nervous system [19]. Neurotransmitters such as acetylcholine and noradrenaline and neurotransmitter candidates such as ATP, CGRP, CCK-8, ENK, GAL, GABA, serotonin, NO, somatostatin, SP, VIP etc have been implicated to different extents in normal and pathophysiological situations. Based on the knowledge gained about the divergent factors controlling the processes of secretion of electrolytes and motility, many interventional strategies have been adopted by researchers and numerous antidiarrheal compounds have been developed but not many compounds are without side effects and therefore there has always been a need for finding new ones.

Acetylcholine is the endogenous neurotransmitter at cholinergic synapses in the central and peripheral nervous system. The stimulation of vagal input to the gastrointestinal tract increases tone, amplitude of contraction and secretory activity of the stomach and intestine. Since such responses are inconsistently seen with administered acetylcholine, possibly because of poor perfusion and rapid hydrolysis by plasma butryl cholinesterase, use of neostigmine was made in the present investigation. Neostigmine is an inhibitor of acetylcholinesterase and increases the amount of acetylcholine at the synapse [3] and thus exerts a pro-kinetic effect. The results show that the *Lantana camara *leaf powder and LCME significantly reduced the % intestinal transit in a dose dependent manner. Lantadene A also produced a statistically significant reduction in % intestinal transit.

The induction of diarrhea with castor oil results from the action of ricinoleic acid formed from hydrolysis of its triglyceride in the oil [20,21]. The released ricinoleic acid produces changes in the transport of water and electrolytes resulting in a hypersecretory response and speeds intestinal transit [3]. The involvement of nitric oxide from neurons in the diarrhea induced by the castor oil has also been proposed [22]. Castor oil increases the induction of prostaglandins [23], causes changes in the permeability and mucosal injuries and stimulates PAF [24] biosynthesis which may result in inflammation of intestinal mucosa. The preventive administration of LCME was associated with significant protection against diarrhea induced by castor oil in mice. *Lantana camara *might possess some compounds with antisecretory properties which may account for its efficacy against diarrhea induced by castor oil in mice.

*Lantana camara *has been reported to be toxic to grazing animals such as cow, buffaloes, sheep and goats [7,25] and laboratory animals such as guinea pigs [8] and female rats [26]. In spite of its widespread toxicity in the *Lantana *affected animals, various parts of this plant have been used in the traditional medicines for treating cuts, ulcers, swelling, eczema, inflammation, fever etc [8]. Gastrointestinal stasis, ruminal stasis, constipation, discolorization of conjunctiva, photosensitization, decreased bile flow and urinary retention in the *Lantana *poisoned animals has been noticed [27-29]. These symptoms resembled those due to atropine toxicity i.e., anticholinergic excess [30,31].

Anti-dysenteric and anti-diarrheal properties of medicinal plants have been suggested to be due to tannins, alkaloids, saponins, flavonoids, sterols and triterpenes and reducing sugars [32]. The sesquiterpene lactones have been reported to have the ability to relax smooth muscles and thereby relieve gastrointestinal disorders [33]. The phytochemical analysis of the *Lantana camara *leaf extract has earlier been shown to contain flavonoids [34], terpenes [35] and their derivatives and pentacyclic triterpenoids [36]. These constituents may mediate the anti-diarrheal action of the *Lantana camara *extract. A verbascoside [37] isolated from *Lantana camara *has been shown to be an inhibitor of protein kinase C. The role of this enzyme has been demonstrated in signal transduction, inflammation and smooth muscle contraction [38] and an inhibition of its activity by a constituent of *Lantana camara *shall result in decrease in motility. Although the anti-diarrheal properties of the reported active terpenoids are well established, aspects of their mechanism of action remain poorly understood. Terpenes, flavonoids and terpenoid derivatives may act by inhibiting release of autocoids and prostaglandins [39,4] thereby inhibit the motility and secretion induced by neostigmine. Intestinal motility alterations in *Lantana camara *foliage poisoned sheep has been described by Pass et al. [40] but no mechanism has been suggested.

## Conclusion

The remarkable antimotility effect of *Lantana camara *methanolic extract against neostigmine as promotility agent points towards an anticholinergic effects due to *Lantana camara *constituents and attest to its wide range of utility in secretory and functional diarrheas and other gastrointestinal disorders in the folklore. This effect was further confirmed by significant inhibition of castor oil induced diarrhea in mice by various doses of LCME. Whatever may be the mechanism of action, LCME may be useful in a wide range of diarrheal states due to disorders of intestinal transit and secretion. Further studies with purified constituents are needed to completely understand the mechanism of anti-diarrheal action of *Lantana camara*.

## Methods

### Plant material and preparation of extract (LCME)

Fresh leaves of *Lantana camara *(red variety) were collected from Palampur (Himachal Pradesh, India). The air-dried, pulverized leaves (100 g) were then exhaustively extracted with methanol (800 ml). The extract was treated with 20 g of activated charcoal and evaporated under reduced pressure. The semi-solid residue (10% yield, w/w) obtained was blackish brown in color, henceforth called *Lantana camara *methanolic extract (LCME). Different doses of LCME (125–1000 mg/kg) and lantadene A (85 and 170 mg/kg, i.p.) were prepared in 0.25% carboxy methyl cellulose (CMC) just before use. The injection volume for each treatment varied from 0.2–0.3 ml depending upon the weight of the animal. The control group animals were given equivalent volumes of 0.25% CMC.

### Preparation of lantadene A

Lantadene A was prepared by method of Barton et al. [13] and its purity (94%) was determined by the method of Sharma et al. [14].

### Animals and treatments

Male mice (laca strain) weighing 20–25 g were obtained from central animal house of Panjab University, Chandigarh and were housed in polypropylene cages under hygienic conditions for one week for acclimatization. The animal ethics committee of Panjab University had approved the study protocol of this project. The animals were given the following treatments:

#### Control

This group received standard pellet diet (Ashirwad Industries, Chandigarh, India) and was given 0.25% CMC, 30 minutes before charcoal meal test.

#### *Lantana camara *leaf powder treated

The animals from this group received leaf powder orally (1% of feed) for 10 days prior to the assessment of intestinal motility.

#### LCME treated

The animals from this group received a single dose of LCME (125, 250, 500 and 1000 mg/kg, i.p.), 30 min before charcoal administration.

#### Lantadene A treated

A single dose of lantadene A (85 and 170 mg/kg, i.p.) was injected.

#### Neostigmine treated

Neostigmine obtained from Tablets (India) Limited, Chennai, India was administered subcutaneously (1 μg/kg) in normal saline.

#### Neostigmine + LCME treated

This group consisted of two subgroups:

Neostigmine (1 μg/kg, s.c.) and LCME (1 g/kg, i.p.) was administered to one subgroup.

The second subgroup received neostigmine (1 μg/kg, s.c.) and LCME (500 mg/kg, i.p.)

The animals were fasted for 24 hours prior to the experiment but permitted water *ad libitum*. On the day of the experiment, treated groups received LCME, lantadene A, neostigmine, neostigmine + LCME.

After 30 minutes of having given the doses as described above, intestinal motility was assessed by orally administrating semisolid test charcoal meal (0.3 ml per mouse) consisting of 10% charcoal and 5% gum acacia. The animals were sacrificed 30 minutes later. The abdomen was opened and the entire small intestine starting from the pyloric end was removed and placed on the blotting paper. The distance traveled by charcoal was measured and expressed as percent intestinal transit [15].



### Castor oil induced diarrhea

Mice were divided into five groups of four animals each, diarrhea was induced by administering 1 ml of castor oil (Qualikems Fine Chemicals Pvt. Ltd. New Delhi, India) orally to mice. Group 1 served as control (2 ml/kg, i.p. saline), groups 2, 3, 4 and 5 received LCME (125, 250, 500 and 1000 mg/kg, i.p.) 1 h before castor oil administration. The number of both wet and dry diarrheal droppings was counted every hour for a period of 4 h and was compared with that of the positive control animals [16].

### Statistical analysis

The results are represented as mean ± S.D. Dunnett's test was used for the evaluation of data and P < 0.01 accepted as significant.

## Abbreviations

LCME: *Lantana camara *methanolic extract GIT: Gastro-intestinal tract, VIP: vasoactive intestinal peptide, GABA: gamma aminobutyric acid, CCK8: cholecystokinin octapeptide, CGRP: calcitonin gene-related peptide, GAL: galanin, SP: substance P, ENK: Enkephalin.

## Competing interests

The author(s) declare that they have no competing interests.

## Authors' contributions

LS and RS were responsible for practically carrying out the experiments

SO – supervised the design and co-ordination of the study

## Pre-publication history

The pre-publication history for this paper can be accessed here:


